# Association of food-hygiene practices and diarrhea prevalence among Indonesian young children from low socioeconomic urban areas

**DOI:** 10.1186/1471-2458-13-977

**Published:** 2013-10-19

**Authors:** Rina Agustina, Tirta P Sari, Soemilah Satroamidjojo, Ingeborg MJ Bovee-Oudenhoven, Edith JM Feskens, Frans J Kok

**Affiliations:** 1 SEAMEO RECFON (Southeast Asian Ministers of Education Organization Regional Center for Food and Nutrition), P.O. Box 3852, Jakarta 10038, Indonesia; 2 Department of Nutrition, Faculty of Medicine, University of Indonesia, Jakarta, Indonesia; 3 Division of Human Nutrition, Wageningen University, Wageningen, The Netherlands; 4 Top Institute Food and Nutrition, Wageningen, The Netherlands; 5 NIZO Food Research, Ede, The Netherlands

**Keywords:** Food-hygiene practices, Young children, Diarrhea, Urban low socioeconomic areas, Indonesia

## Abstract

**Background:**

Information on the part that poor food-hygiene practices play a role in the development of diarrhea in low socioeconomic urban communities is lacking. This study was therefore aimed at assessing the contribution of food-hygiene practice to the prevalence of diarrhea among Indonesian children.

**Methods:**

A cross-sectional study was conducted among 274 randomly selected children aged 12–59 months in selected low socioeconomic urban areas of East Jakarta. The prevalence of diarrhea was assessed from 7-day records on frequency and consistency of the child’s defecation pattern. Food-hygiene practices including mother’s and child’s hand washing, food preparation, cleanliness of utensils, water source and safe drinking water, habits of buying cooked food, child’s bottle feeding hygiene, and housing and environmental condition were collected through home visit interviews and observations by fieldworkers. Thirty-six practices were scored and classified into poor (median and below) and better (above median) food-hygiene practices. Nutritional status of children, defined anthropometrically, was measured through height and weight.

**Results:**

Among the individual food-hygiene practices, children living in a house with less dirty sewage had a significantly lower diarrhea prevalence compared to those who did not [adjusted odds ratio (OR) 0.16, 95% confidence interval (CI) = 0.03-0.73]. The overall food-hygiene practice score was not significantly associated with diarrhea in the total group, but it was in children aged < 2 years (adjusted OR 4.55, 95% CI = 1.08-19.1).

**Conclusions:**

Overall poor mother’s food-hygiene practices did not contribute to the occurrence of diarrhea in Indonesian children. However, among children < 2 years from low socioeconomic urban areas they were associated with more diarrhea.

## Background

Despite the substantially declining mortality rate from diarrhea in developing countries, diarrhea still accounts for approximately 11% of all mortality in children under 5 years of age [1]. Diarrhea incidence rates among children in this age group in developing countries including Indonesia has declined in the past 20 years, but the burden of disease has remained consistent with respect to age [2].

Diarrhea incidence remains a tremendous burden on children in low- and middle-income countries [2] due to multiple determinants [3] such as child malnutrition [4], low socioeconomic status and education of mothers [5,6], lack of safe drinking-water, inadequate sanitation and poor hygiene [7,8], crowding [9] and low maternal age [10]. These determinants of diarrheal disease are strongly linked to poverty and social inequities [11]. Furthermore, diarrheal incidence is highest in the first two years of life and declines as a child grows older [12].

Although the determinants of diarrhea among children are well described, information on the part of food-hygiene practices play a role in the development of diarrhea and malnutrition among children in low socioeconomic urban communities is lacking [13]. Many studies conducted in the urban settings of developing countries focused on the risk factors of diarrhea related to environmental conditions and utilization of sanitation facilities [14-16]. A previous study on food-hygiene missed some important practices such as food storage, thorough cooking and adequate holding temperature [13] as recommended by the World Health Organization (WHO) [17]. Mothers and children in low socioeconomic urban areas in East Jakarta with limited hygiene and sanitation facilities tend to have poor hygiene practices such as using dirty cooking or eating utensils for their children [18]. While poor hygiene practices, especially in food preparation and feeding practices, may increase the risk of having diarrhea, up to 70% of diarrhea episodes are actually caused by water and food contaminated with pathogens [19]. Therefore, we hypothesized that the prevalence of diarrhea and malnutrition among children in low socioeconomic urban areas of East Jakarta is high, not only because children and mothers are exposed to the environmental factors that cause diarrhea such as unsafe water, and poor sanitation and hygiene, but also due to poor food-hygiene practice. To address this hypothesis, we assessed the association of food-hygiene practice with the occurrence of diarrheal disease among under-five children in selected urban low socioeconomic areas of East Jakarta, Indonesia. The results of this study can be useful in designing an intervention study, health plans and policies related to mother and child hygienic behavior.

## Methods

### Study design

A cross sectional study was carried out from October 2004 to February 2005 in an urban area of Jatinegara, East Jakarta district, Indonesia. This district was selected because it has the highest prevalence of diarrhea and underweight (24%) in under-five children in Jakarta province, based on passive surveillance of the local government. In this urban area, purposive sampling was used to target low socioeconomic households: those in which the housing location was along the river side (flooding area) and households within specific low socioeconomic areas of the city centre (non-flooding area).

### Subjects

A total of 274 children aged 12 – 59 months were selected randomly from the community registry. Only the youngest child who was selected for the study if a family had more than one child at this age category. At the time of selection, the children had been living in this district for at least 6 months. Informed consent was obtained from mothers or caregivers after they had received an explanation about the study’s objective and method. The study protocol was reviewed and approved by the Ethical Committee of the Faculty of Medicine, University of Indonesia and National Center for Research and Development, Ministry of Health Republic of Indonesia.

### Data collection

Data collection in this study was divided into three phases: (a) interview: socio-demographic factors of the family, general characteristic of children and mother, breastfeeding practices, utilization of health services, mother’s and child’s hand washing, food preparation, cleanliness of utensils, water source and safe drinking water, habits of buying cooked food, and child’s bottle feeding practices, (b) observation: the child's defecation and diarrhea pattern to obtain information on diarrhea prevalence, house and environmental condition, food storage, water source and drinking water storage, and (c) anthropometric measurements.

A structured questionnaire, used for conducting interviews and observations, consisted of four sections: (a) general information, (b) diarrhea prevalence, (c) complete list of food-hygiene practices, and (d) nutritional status. The questionnaire was developed based on a similar survey, the guidelines set by the WHO, information from staff members of the community health centers and voluntary mothers, and two group discussions in two chosen villages with 8–10 mothers aged less than 45 years old, having children between 1–5 years of age, living in the study area.

Mothers were asked to fill in the form according to what she had observed in her child's defecation pattern (time, frequency and stool’s visual appearance) [20] for seven days, starting at 8.00 AM each day. Every day one field worker collected the observation form of defecation pattern. The field worker checked whether the child being observed had diarrhea or not based on the mother’s record.

In addition, the interview was conducted by the field worker to the mothers or caretakers in their house after the anthropometric measurement of their child. Mothers or caregivers were interviewed on the mother’s and child’s hand washing, food preparation, cleanliness of utensils, water source and safe drinking water, habits of buying cooked food, child’s bottle feeding hygiene and housing and environmental condition using a structured questionnaire. During the interview, the field worker observed the house and surrounding condition, sewage condition, availability of a latrine, food storage, water source and drinking water storage.

### Diarrhea prevalence

Diarrhea was defined as defecation frequency of three or more loose/liquid stools in a day [21]. We calculated the period prevalence of diarrhea as the percentage of children suffering from diarrhea assessed during a 7-day recording period. Recall periods of more than 48 hours may lead to underreporting of diarrhea cases [22] therefore we chose recording diarrhea during a 7-day period to avoid information bias in diarrhea prevalence and a 7-day recall period is commonly used in practice [23]. Many studies defined diarrhea prevalence based on a recall of mother 2 weeks or 3 months before the study.

### Food-hygiene practice

All 36 variables that may contribute to food-hygiene practices, i.e. mother’s hand washing before preparing food and feeding the child, child’s hand washing before eating a meal and after defecating/urinating, food preparation, cleanliness of utensils, water source and safe drinking water, habits of buying cooked food, child’s bottle feeding hygiene and housing and environmental condition, were summed up into a total of 36 scores. Each variable was scored as 0 or 1, with 1 representing a positive practice [24]. The positive practice was judged by field worker based on pre-instruction about the undesirable/favorable practices. All individual variables were assigned equal importance (Table 1). Total composite score was classified into poor and better food-hygiene practice based on the median score of the population, i.e. food-hygiene practice score ≤ 19 was considered as being poor (n = 168), while a score > 19 was considered as being better practice (n = 168).

**Table 1:** Scoring of food-hygiene practices

**Food-hygiene practices**		** Score**	**Methods of measurement**
	**Undesirable response = 0**	**Favorable response = 1**	
*House and environmental condition*			
Clean inside the house	Dirty	Clean	Observation
Clean surrounding the house	Many garbage	Clean	Observation
Existence and less dirty sewage	Dirty or no sewage system	Less dirty	Observation
Existence of latrine	No	Yes	Observation
Latrine with closet and septic tank	no closet and/or septic tank	with closet and septic tank	Observation
Child’s feces was thrown in latrine	No	Yes	Interview
Other family defecated in latrine	No	Yes	Observation
Closed domestic waste disposal	Open	Close	Interview
*Mother’s hand washing*			
Before preparing the food	No	Yes	Interview
Before feeding the child	No	Yes	Interview
Using water and soap	With water only	With water an soap	Interview
*Child’s hand washing*			
Before eating a meal	No	Yes	Interview
After defecating or urinating	No	Yes	Interview
Using water and soap	With water only	With water an soap	Interview
*Food preparation*			
Source of food	Bought from outside	Cooked by mother	Interview
Cooked special food for child	No	Yes	Interview
Food holding time before eaten	≥ 1 hour	< 1 hour	Interview
Reheating the food before child eating	Never or only once	Every time the child wanted to eat	Interview
Feeding child with warm or hot food	Cold	Warm or hot	Interview
Store food covered or closed	Open	Covered or closed	Observation
*Cleanliness of utensils*			
Place to wash utensils	Outside	Inside	Interview
Wash utensils with flowing tap water	No, collect the water in the pail	Yes	Interview
Frequency of changing water in the pail	≤ 3 times in a day	Every time wanted to wash the dishes or > 3 times in a day	Interview
*Water source and safe drinking water*			
Piped water source	Outdoor or ground water	Piped water	Interview
Existence of septic tank	Not available	Available	Observation
Water treatment for drinking water	Boiling water	Refill or branded drinking water	Interview
Cover on water storage	Open	Covered or closed	Observation
*Habits of buying cooked food*			
Frequency of buying street food	Frequent	Not frequent	Interview
Bought hot food from outside	No, cold food	Yes, hot food	Interview
Food given to child were still hot	No	Yes	Interview
If the food still hot, food directly eaten	No	Yes	Interview
Not frequently bought snack	Frequent	Not frequent	Interview
Type of bought package	Non package snack	Package snack	Interview
*Child’s bottle feeding hygiene (only for bottle fed children)*			
Using bottle feeding	Yes, with bottle milk	No, with glass or no bottle feeding	Interview
Bottle was washed and boiled	Washed only	Washed and boiled or no bottle feeding	Interview
Clean bottle milk	Less clean	Clean or no bottle milk	Interview

### Nutritional status

Wasted, underweight and stunted were defined as weight-for-height z-score (WHZ), weight-for-age z-score (WAZ), and height-for-age z-score (HAZ) of less than – 2 SD based on nutritional indices of the WHO Child Growth Standard, respectively [25]. Children were weighed lightly clothed without shoes using an electronic scale (SECA platform 770, SECA, Hamburg) with a precision of 0.1 kg. For children who were not able to stand, the child’s weight was obtained by subtracting the mother’s weight from the measured weight. Body stature was measured using a microtoise for children who could stand erectly, with a precision of 0.1 cm [26]. While for children who could not stand, a SECA length board was used.

### Feeding practices of children

Feeding practices of children were assessed by interviewing the mothers on history of breast feeding status, bottle feeding and snacking practices among their children. We classified the breastfeeding status of children as early initiation (received breastfeeding immediately after birth and received colostrum at birth), exclusive (received exclusive breastfeeding for 6 months period and no pre-lacteal feeding at birth), continuation of breastfeeding (current status and duration of breastfeeding). These classifications of breastfeeding status were based on retrospective data, except for the continuation of breastfeeding that was based on current status data (more applied for child age’s < 36 months) [15]. Bottle feeding practice was assessed as a current status of child who is fed milk with a bottle [15]. Snacking practices referred to current practice of frequency of buying and type of snack.

### Statistical analysis

The minimum study sample size was calculated for estimating the actual prevalence of diarrhea among areas under study. The anticipated proportion of 15% was chosen as average diarrheal prevalence in East Jakarta. With 5% precision and anticipating on 20% missing data, the minimal sample was 245 children.

We used STATA for windows release 11 (College Station, Texas 2009) for data analyses. Data on weight and height of the children were transferred into z-scores using WHO software [25].

Variables, such as child’s age, sex, area of living, nutritional status, breastfeeding practices, utilization of health services, maternal schooling, socioeconomic status (SES), family size and number of under-five children living under the same roof that had a *p* < 0.25 based on bivariate analysis by *X*^*2*^ test were considered as potential confounders [27]. SES was categorised based on the criteria of the local government (ownership of the house, monthly income, monthly expenditure, type of floor, and availability of latrine). The average score of SES in the study population was 15. SES was classified into a binary category as described in another study [28]: households with a median score or lower (≤15) as very low SES; or above median score (>15) as medium-low SES.

We next performed single and multiple logistic regression models and used these models to assess the association between diarrhea and feeding practices. First, we calculated the unadjusted odds ratio (OR) and 95% confidence intervals (CI) of each variable using bivariate analysis. If the 95% CI did not include one, i.e. *p* < 0.05, we considered the result statistically significant. Secondly, all potential covariates were included in logistic analysis to estimate the adjusted OR and 95% CI. Thus, a multiple logistic regression model was used to account for the effect of several potential confounding factors, i.e., age, weight-for-height z-score, immunization status, family size and number of under-five living under the same roof. Logistic regression was also used to assess whether there was any effect modification (*p* < 0.05, test homogeneity of the OR). Finally, we assessed the unadjusted and adjusted OR and 95% CI of two classifications of feeding practices versus prevalence of diarrhea.

## Results

More than half of the children resided in the flooding area along the river side. One third of children were from families consisting of six members (Table 2). Average age of mothers was 30 years while median age of fathers was 34 years (range 20 – 66 years). The main occupation of fathers was private sectors employee (34%), street vendor, small-scale trader or self-employed (29%), driver (12%), construction, factory and day laborer (17%), government employee (3%), while 5% was unemployed. The majority of mothers (83%) were housewives. A few mothers worked as small traders, street vendors, laborers and launderers.

**Table 2:** General characteristics of the study population in Jatinegara sub-district, East Jakarta (n = 274)

**Variables**	**n (%) or median (min-max)**
Area of living:	
Flooding site	168 (61%)
Non flooding site	106 (39%)
Children	
Median age (months)	31.1 (12.1 – 59.8)
Age group (months)	
12 – 23	93 (34%)
24 – 35	71 (26%)
36 – 47	65 (24%)
48 – 59	45 (16%)
Gender	
Boy	151 (55%)
Girl	123 (45%)
Mother	
Median age (years)	30 (17–50)
Length of schooling (n = 273)^a^	
≤9 years	156 (57%)
>9 years	116 (43%)
Household	
Nuclear or extended family^b^	143 (52%) or 131 (48%)
Family size	6 (3–22)
Socioeconomic status^c^	
Medium – low	125 (46%)
Very low	149 (54%)

^a^One mother was illiterate.

^b^Nuclear family consists of only father, mother and children; extended family consisted of a nuclear family and their close relatives living under the same roof.

^c^Categorised based on total score of socioeconomic status (SES) criteria of local government (ownership of the house, monthly income, monthly expenditure, type of floor, and availability of latrine).

The minimum labor salary rate of Rp. 600,000 (US$ 65) per month was used to determine the level of family income and expenditure. One third of the families had monthly income of < US$ 65 indicating that they belonged to a very low-income group. In a second third of the families, the monthly income was between US$ 65 – 110 indicating the low income and expenditure group. The last third was the medium income and expenditure group, with a monthly income > US$ 110 (data not shown).

Diarrhea prevalence of 10% was observed during a 7-day record (Table 3). There was no significant difference in diarrhea prevalence between children living in flooding and non-flooding areas. The highest diarrhea prevalence (17%) was found in children aged 12 to 23 months. Prevalence tended to decrease as child’s age increased, and the difference in the prevalence was significant between children aged 12 to 23 months and older. Diarrhea prevalence was not significantly different between sexes. The prevalence of stunting, underweight and wasting among children aged 12 – 59 months was 32%, 19% and 12%, respectively, and not different between age groups, sexes and living areas.

**Table 3:** Prevalence of diarrhea and malnutrition by age and sex of under-five children in Jatinegara sub-district, East Jakarta

**Variables**	**n**	**Diarrhea prevalence (%)**^**a**^	**Undernutrition prevalence (%)**		
			**Stunted (HAZ < -2)**	**Underweight (WAZ < -2)**	**Wasted (WHZ < -2)**
Area of living					
Flooding	168	10.1	36.3	26.8	9.5
Non flooding	106	10.4	25.5	17.9	9.5
Age group (months)					
12 – 23	93	17.2^*^	32.3	19.4	11.8
24 – 35	71	8.5	32.4	25.4	8.6
36 – 47	65	6.2	33.8	24.6	6.2
48 – 59	45	4.4	28.9	26.7	11.1
All ages	274	10.2	32.1	23.4	9.5
Gender					
Boy	151	9.9	35.8	25.2	11.9
Girl	123	10.6	27.6	21.1	6.6

*HAZ*, height-for-age z-score; *WAZ*, weight-for-age z-score; and *WHZ*, weight-for-height z-score.

^a^Weekly point prevalence, assessed from a 7-day records.

^*^Significantly different between ages, *p* < 0.05, *X*^2^ test.

Factors found to be significantly associated with diarrhea were age, weight-for-height z-score, immunization status, family size and number of under-five children living under the same roof (Table 4). The odds ratio for children aged < 2 years was 2.9 times (95% CI = 1.32-6.48). Children with complete immunization were protected from diarrhea (OR 0.35, 95% CI = 0.16-0.78). Children belonging to families with ≥ 6 household members had 2.3 times higher risk of suffering from diarrhea (95% CI = 1.03-4.98). Presence of >1 child under five years of age living in the same household increased the risk of suffering from diarrhea 2.8 times (95% CI = 1.26-6.16). The risk of diarrhea was 1.6 times higher when children had a mother with low education level and family with very low SES, but this was not significantly different, nor was the association with wasting (OR 2.3, 95% CI = 0.70-6.73).

**Table 4:** Risk factors associated with the occurrence of diarrhea in children aged 12 – 59 months (n = 274)

**Risk factors**	**Total n**	**Diarrhea**	
		**%**	**Unadjusted OR (95% CI)**
Children			
General characteristic			
Living in flooding area	168	10	0.97 (0.44–2.17)
Age ≤ 2 years	93	17	2.93 (1.32–6.48)^*a^
Age ≤ 3 years	164	13	2.69 (1.05–6.86)^*a^
Boy	151	10	0.93 (0.43–2.04)
Nutritional status			
Wasted	36	19	2.32 (0.70–6.73)
Underweight	63	14	1.67 (0.71–3.89)
Stunted	88	8	0.68 (0.28–1.66)
Breastfeeding practice history			
Received breastfeeding after birth (n = 273)	170	12	1.58 (0.67–3.74)
Received colostrum at birth	215	10	1.01 (0.39–2.61)
Received exclusive breastfeeding or no pre-lacteal feeding at birth^†^	103	8	0.64 (0.27–1.50)
Breastfeeding duration ≥12 months (n = 273)	224	9	0.62 (0.25–1.55)
Utilization of health services			
Received vitamin A supplementation (n = 273)	224	9	0.62 (0.25–1.55)
Received complete immunization	188	7	0.35 (0.16–0.78)^*a^
Mother			
Maternal schooling ≤ 9 years (less or equal to junior high school) (n = 273)	157	12	1.64 (0.71–3.76)
Household condition			
Socioeconomic status: very low	149	12	1.58 (0.70–3.56)
Family size ≥ 6 persons	98	15	2.27 (1.03–4.98)^*b^
Under-five children living under the same roof >1	79	18	2.78 (1.26–6.16)^*b^

^†^Prelacteal feeding was any nonhuman milk food or fluids provided to the new-borns before breastfeeding on the first day of life [29].

^*a^Statistical significant at *p* < 0.05, *X*^2^ test.

^*b^Statistical significant at *p* < 0.05, Fisher’s exact test.

Among the individual food-hygiene variables, children who lived in the house with less dirty sewage had significantly lower diarrhea prevalence compared to those who did not (adjusted OR 0.17, 95% CI = 0.04-0.75) (Table 5). Children whose mother had habits of buying package snacks showed significantly higher diarrhea prevalence compared to those who did not (unadjusted OR 3.00, 95% CI = 1.01-8.93). However, the risk of diarrhea became non-significant after adjustment for age, weight-for-height z-score, immunization status, family size and number of under-five children living under the same roof.

**Table 5:** Distribution of diarrhea prevalence by determinant factors of food-hygiene practices among children aged 12 – 59 months

**Food-hygiene practices**^**a**^	**Total n**	**Diarrhea**				
		**%**	**Unadjusted OR (95% CI)**	***p*-value**	**Adjusted OR (95% CI)**^**b**^	***p*-value**
*House and environmental condition (n = 274)*						
Clean inside the house	134	7	0.46 (0.20–1.05)	0.07	0.68 (0.27–1.68)	0.40
Clean surrounding the house	139	8	0.60 (0.27–1.33)	0.21	0.69 (0.29–1.64)	0.40
Existence and less dirty sewage	74	3	0.19 (0.04–0.80)^*^	0.02^*^	0.16 (0.03–0.73)^*^	0.02^*^
Existence of latrine	172	12	1.89 (0.77–4.61)	0.16	1.53 (0.62–3.92)	0.37
Latrine with closet and septic tank	105	13	1.70 (0.78–3.73)	0.18	1.43 (0.62–3.29)	0.41
Child’s faeces was thrown in latrine	138	7	0.51 (0.23–1.15)	0.11	0.62 (0.27–1.46)	0.28
Other family defecated in latrine	241	11	1.87 (0.42–8.29)	0.41	1.63 (0.35–7.58)	0.53
Closed domestic waste disposal	145	10	0.87 (0.40–1.90)	0.73	0.96 (0.42–2.21)	0.93
*Mother’s hand washing (n = 274)*						
Before preparing food	80	9	0.79 (0.32–1.94)	0.61	0.79 (0.31–2.04)	0.62
Before feeding the child	240	11	4.18 (0.55–31.8)	0.17	4.16 (0.53–32.8)	0.18
Using water and soap	193	9	0.73 (0.32–1.66)	0.45	0.71 (0.30–1.70)	0.45
*Child’s hand washing (n = 274)*						
Before eating a meal	185	10	1.02 (0.44–2.35)	0.97	1.08 (0.45–2.61)	0.86
After defecating or urinating	49	10	1.00 (0.36–2.77)	0.99	1.14 (0.39–3.32)	0.81
Using water and soap	154	10	0.89 (0.41–1.95)	0.77	0.87 (0.38–1.99)	0.74
*Food preparation (n = 274)*						
Food cooked by mother	201	10	1.10 (0.45–2.71)	0.84	1.00 (0.38–2.62)	0.99
Cooked special food for child^c^	75	16	2.48 (0.99–6.19)	0.05	2.56 (0.88–7.58)	0.08
Food holding time before eaten <1 hour	151	10	0.93 (0.43–2.04)	0.86	0.78 (0.34–1.78)	0.55
Reheating the food before child eating^d^	191	9	0.46 (1.18–1.13)	0.09	0.48 (0.18–1.28)	0.14
Feeding child with warm or hot food	150	11	1.31 (0.59–2.92)	0.50	1.16 (0.49–2.74)	0.74
Store food covered or closed	254	10	1.03 (0.23–4.67)	0.97	0.93 (0.19–4.41)	0.92
*Cleanliness of utensils (n = 274)*						
Wash utensils inside house	236	11	2.23 (0.51–9.80)	0.29	1.97 (0.43–9.11)	0.38
Wash utensils with flowing tap water	76	9	0.86 (0.35–2.10)	0.73	0.86 (0.33–2.21)	0.75
Change water in the pail	225	9	0.50 (0.21–1.21)	0.13	0.48 (0.18–1.26)	0.14
*Water source and safe drinking water(n = 274)*						
Piped water source	72	10	0.93 (0.38–2.29)	0.87	0.97 (0.37–2.51)	0.94
Existence of septic tank	106	13	1.67 (0.76–3.67)	0.20	1.41 (0.61–3.25)	0.42
Refill or branded drinking water source	57	11	1.04 (0.40–2.71)	0.93	1.02 (0.37–2.80)	0.98
Cover on water storage	270	10	1.32 (0.37–4.76)	0.67	1.06 (0.28–4.07)	0.93
*Habits of buying cooked food (n = 274)*						
Not Frequently bought street food	109	7	0.57 (0.24–1.35)	0.21	0.52 (0.21–1.29)	0.16
Bought hot food from outside	159	13	1.92 (0.82–4.54)	0.14	1.71 (0.69–4.26)	0.25
Food given to child were still hot	235	11	1.43 (0.41–4.98)	0.58	1.48 (0.38–5.73)	0.57
If the food still hot, food directly eaten	193	9	0.61 (0.27–1.38)	0.24	0.71 (0.30–1.67)	0.43
Not frequently bought snack	29	17	2.01 (0.70–5.77)	0.19	1.43 (0.43–4.75)	0.56
Bought package snack	188	13	3.00 (1.01–8.93)*	0.048	2.37 (0.76–7.35)	0.14
*Child’s bottle feeding hygiene*						
Using bottle feeding (n = 155)	96	13	2.67 (0.71–9.88)	0.14	3.33 (0.78–14.2)	0.10
Bottle was washed and boiled (n = 96)	24	8	0.56 (0.11–2.78)	0.48	0.67 (0.11–3.97)	0.66
Clean bottle milk (n = 95)	32	6	0.35 (0.07–1.72)	0.20	0.22 (0.03–1.53)	0.13

Note: OR, odd ratio.

^a^Variables are categorical and indicate favorable practice, except for the variable of 'using bottle feeding” that was undesired practice.

^b^Adjusted for age, weight-for-height z-score, immunization status, family size and number of under-five children living under the same roof.

^c^n = 201; ^d^ n = 234.

^*^Statistical significant at *p* < 0.05, *X*^2^ test.

The association between the overall food-hygiene practice score and diarrhea among children aged 12 – 59 months was not significant, neither in the crude nor in the adjusted analyses (Table 6). However, a significant interaction with age and food-hygiene practices was observed (*p* < .001). Stratified analysis by age showed that a poor food hygiene score was independently associated with diarrhea in children < 2 years (*p* < .05), although the confidence interval was very wide. Because sewage condition and water sources may independently associate with diarrhea in children <2 years, we repeated the logistic regression analysis versus feeding practices excluding these variables in a composite score. However, the adjusted OR for poor hygiene practices were not different when these variables were excluded from the composite scores of feeding practices (adjusted OR 4.08, 95 CI = 0.96-17.4; p = .058) (data not shown).

**Table 6:** Association between food-hygiene practices and diarrhea among children aged 12 – 59 months (n = 274)

**Determinants**	**Food-hygiene practice**	**Diarrhea**		
		**%**	**Unadjusted OR (95% CI)**	**Adjusted**^**a **^**OR (95% CI)**
All children	Poor	11	1.15 (0.51–2.60)	1.33 (0.57–3.14)
	Better	9	1.00	1.00
	*p-*value		0.73	0.51
Stratified by age group				
≤ 2 y (n = 93)	Poor	23	2.63 (0.78–8.89)	4.55 (1.08–19.10)^*^
	Better	10	1.00	1.00
	*p-*value		0.12	0.04^*^
> 2 y (n = 181)	Poor	5	0.55 (0.17–1.78)	0.62 (0.18–2.14)
	Better	9	1.00	1.00
	*p-*value		0.32	0.38

Note: Poor food-hygiene practice (score ≤19 of 36 score); good practice (score >19 of 36 score).

^a^Adjusted for age (continuous), weight-for-height z-score (continuous) and number of under-five children living under the same roof (>1/1).

^*^Statistical significant at *p* < 0.05, *X*^*2*^ test.

## Discussion

Our study indicates that the risk of having diarrhea is increased in children aged <2 years whose mother had poor food-hygiene practices. Children who lived in houses with less dirty sewage had a lower risk of diarrhea.

Our finding on diarrhea prevalence is consistent with many studies in developing countries in similar settings such as Vietnam [13], Thailand [30] and Bangladesh [31] confirming that children aged <2 years were more vulnerable to suffer from diarrhea. This finding is also in line with the previous review that diarrheal diseases were extremely high during the weaning period (6–24 months) [19]. Our study was conducted in a rainy season (November – April), which was assumed to be the peak of diarrhea prevalence as described in a previous study within a similar community [32]. However, some recent studies in developing countries observed high diarrhea prevalence in dry seasons [33-37]. Performing the study in a rainy season may lead to an overestimation of diarrhea prevalence because there is a strong link between diarrheal illnesses and weather- and climate-related events. The prevalence of diarrhea in our study in all (10%) or in younger age (17%) corresponds with the known prevalence, thus an effect of season was not clearly seen.

Direct association between food-hygiene practices and diarrhea prevalence in children has been suggested in several epidemiologic studies in developing countries e.g. Vietnam [13], Bangladesh [31,38], Nigeria [39], Nicaragua [40], Brazil [41] and Congo [42], but findings are not conclusive [43]. The practices were evaluated either as combined or individual practices such as hand washing, food preparation and storage, sewage condition, and safe water source and storage. Our present study typically provides a comprehensive and more complete set of local practice identified in our community and presents not only single variables, but also a summary score of practices. This approach may give advantages because important and specific local practices are not missed. By using more complete set of local food-hygiene practices than other studies, our study indicated that the poor food-hygiene practice score was not associated with the prevalence of diarrhea among children under five, but was significantly associated with more diarrhea among children aged <2 years.

We presented 36 single variables as a summary score reflecting a food-hygiene practice index. This index was dichotomized into poor and better food-hygiene practice based on the median score of the population. Some studies with much larger sample sizes classified the total score into tertiles or quartiles. However, this classification was not applied in our study because it leads to very large confidence intervals, due to small numbers, which would complicate the interpretation. This scoring system for food-hygiene practices can be considered as a preliminary step in the development of a methodology to measure and quantify the different types of food-hygiene practices in a community setting.

The increased risk of having diarrhea in children aged <2 years whose mother had poor food-hygiene practices in our study was similarly observed in a peri-urban district of Guinea-Bissau [44]. This finding may be explained by the fact that weaning foods for young children prepared under unhygienic conditions are frequently contaminated with pathogens and are an important risk factor of diarrhea transmission [19]. However, our study was not able to demonstrate an association between the contamination of weaning foods and diarrhea due to a lack of assessment of specific enteropathogens. Although food-borne infection is the main route of transmission of gastrointestinal infections in developed countries, their contribution to the burden of diarrhea in low-income settings is still unclear [43]. Contaminated weaning food has been suggested as a major contributor to diarrhea in low-income settings as up to 70% of diarrhea episodes are actually caused by water and food contaminated with pathogens [19] although observational studies gave inconclusive results [45]. A study in Gambia failed to document an association between water or weaning food contamination and higher rates of diarrheal morbidity [46]. Two recent studies found an increased risk of diarrhea associated with the consumption of maize-based weaning foods [42,46]. However, in one of these studies, this association was only significant in children living in rural communities [42]. Therefore, the association between contaminated weaning foods and diarrheal diseases in young children living in urban setting of developing countries remains lacking [45].

Transmission of Enterotoxigenic *Escherichia coli* (ETEC) is known to be specifically associated with contaminated weaning foods among younger children in developing countries [47,48] and was considered to be responsible for the diarrhea-induced weight faltering [19]. Younger children are also at risk to be infected by rotavirus that is transmitted primarily person-to-person through the fecal-oral route [49]. Children are infected with rotavirus through contact with an infected person outside and in the household and poor food-handling hygiene practices such as the contamination of the mother’s hands by infected fomites or surfaces [50-52]. Improved water and sanitation is not sufficient to reduce the spread of this virus, as indicated by similar rates of illnesses in developed and developing countries [53,54]. The great disparity in mortality associated with diarrhea caused by rotavirus among children in industrialized and developing countries, is likely related to differences in access to appropriate and timely medical care and hydration therapy [54] and a greater prevalence of malnutrition [55].

Several food hygiene practices assessed in our study such as mother and child’s hand washing practice, food preparation (eg. food holding time and reheating food before eaten) and habits of buying cooked food from outside (eg. not frequently buying, food bought when still hot and directly eaten), the use of clean eating or cooking utensils, and the cleanliness of bottled milk were related to the sources of food-borne transmission but did not show statistically significant associations with diarrhea. The best studied hygiene practice with consistent evidence in developing countries is hand washing [43]. Evidence from the randomized trials (RCTs) on hand washing showed reductions in diarrhea of around 30%, and of 43–53% if soap is used [56-58]. Our study found that mother and child who reported washing their hands with water and soap had lower percentage of diarrhea than who did not, however, our study failed to demonstrate statistically significant associations.

The absence of basic sanitation facilities in a low socio-economic family may lead to poor food hygiene and sanitation practices in the households [18,59]. This is because water supply, source of drinking water and house and environmental hygiene were known to be related to either the risk of food- or water-borne transmission [18,60]. Although the human waste disposal and environmental hygiene were poor in our study area [61], we did not find a significant association of diarrhea and the source of water supply and drinking water. This can be explained because the majority of respondents in our study reported boiling their drinking water before consumption, which is in line with the study done by Vollaard and colleagues in an almost similar study site [61], and/or used refill or package water. These practices may apparently prevent the risk of drinking water contamination to the children in this area. Our study confirmed that children who lived in a house with less dirty sewage had significant lower risk of having diarrhea than children who did not. A previous study conducted in an urban poor setting in Indonesia also reported an increased risk of having diarrhea in children with unavailability of sewage and/or a place to dispose the child’s stools [62]. Pathogens in feces disposed in sewage near the house can contaminate the environment and the food eaten by children [7,39,63,64]. Contamination of drinking water by sewage through pump failure or blockage of a sewage system [65] and outbreaks of viral gastroenteritis resulting from sewage contamination of water supplies have been previously described [66,67]. Unfortunately, information on diarrhea-causing pathogens could not be obtained in our study.

Since our study purpose was to obtain global insight into factors (i.e., food hygiene practices) that could additionally contribute to transmission of diarrhea in children, our approach using a rapid survey has several limitations. A 7-day recording period prevalence of diarrhea is commonly used in practice [68] and may avoid information bias. However, this period may be too short to gather information on the number of diarrhea episodes in the household, which is essential to explain within-household transmission. Information on the assessment of intra-household factors (controllable) versus external non-controllable factors (e.g. sewage) or behavioural versus socioeconomic factors or food versus water-associated transmission, recent episode and health care visits due to diarrhea of household members, use of oral rehydration solution during diarrhea, or association between income and sewage condition was missing in our study that might be subject for further investigations.

Although diarrhea prevalence in our study showed a similar trend with other studies, the association between food hygiene practice and diarrhea was not significant. The lack of association may be due to the method used for data collection, study design or selection of food hygiene practices. We relied on recall methods to investigate food-hygiene practices. This may be the reason why we could not observe significant associations between overall and some of the single food-hygiene practices. Mothers may report socially desirable practices which they do not perform or forget some of the practices. However, we observed that mothers in our study were very motivated and spontaneously answered the questions without hesitation or interferences from other people. The lack of association between hand washing and diarrhea in our study may be due to application of single report instead of repeated observation methods to assess this practice. Repeated observations can avoid risks of misclassifying exposure that reduces the statistical power to identify associations [38]. The RCTs method of other studies [56-58] provide much stronger causal relationship. Some data in this study derived from direct observation, such as observations in the house and its environment, were significantly associated with diarrhea prevalence. Direct observation is most preferred because it allows first hand data collection in a natural setting as used in prospective cohort studies in Nigeria and Brazil [40,41]. Although there can be potential bias in (any) recall method, this way of data collection is less expensive and relatively fast, and is still considered as a way to find out the extent of the problem in a poor community [24].

## Conclusions

In conclusion, the poor food-hygiene practice score was not associated with the prevalence of diarrhea among children under five, but was significantly associated with more diarrhea among children aged <2 years. Therefore, food safety education should be especially targeted to this age group, focusing on good food-hygiene practices and less dirty sewage. Direct observation methods are preferred for future studies to avoid over- or under-reporting by the mothers on their child's feeding practice. Other typical local food hygiene practices (eg. habits of eating with hands, sharing food from the same plates) should be explored in the future research.

## Competing interests

The authors declare that they have no competing interests.

## Pre-publication history

The pre-publication history for this paper can be accessed here:

http://www.biomedcentral.com/1471-2458/13/977/prepub
